# Molecular mechanisms and targeted therapeutic strategies for glycolytic reprogramming in osteosarcoma

**DOI:** 10.1016/j.gendis.2026.102111

**Published:** 2026-02-24

**Authors:** Xuewen Ni, Ping Xia, Mengxue Liang, Li Zhang, Jiaqi Chen, Zhuojia He, Hua Huang, Jing Feng, Xun Niu, Feifei Pu

**Affiliations:** a First Clinical College, Hubei University of Chinese Medicine, Wuhan, Hubei 430060, China; b Department of Orthopedics, Wuhan Fourth Hospital, Wuhan, Hubei 430030, China; c Department of Orthopedics, Traditional Chinese and Western Medicine Hospital, Hubei University of Chinese Medicine, Wuhan, Hubei 430022, China; d Department of Orthopedics, Wuhan No.1 Hospital, Wuhan, Hubei 430022, China; e Department of Orthopedics, Traditional Chinese and Western Medicine Hospital of Wuhan, Tongji Medical College, Huazhong University of Science and Technology, Wuhan, Hubei 430022, China; f Henan Luoyang Orthopedic Hospital (Henan Provincial Orthopedic Hospital), Henan University of Chinese Medicine, Zhengzhou, Henan 471002 China; g Department of Otorhinolaryngology, Union Hospital, Tongji Medical College, Huazhong University of Science and Technology, Wuhan, Hubei 430022, China

**Keywords:** Chemoresistance, Glycolysis, Lactylation, Metabolic reprogramming, Osteosarcoma, Therapeutic targeting

## Abstract

Osteosarcoma, a highly invasive and metastatic malignant bone tumor, has a poor prognosis primarily because of early pulmonary metastasis and resistance to conventional treatments. The malignant progression of osteosarcoma cells significantly depends on glycometabolic reprogramming, a process characterized by enhanced aerobic glycolysis. This review begins by mapping the multi-dimensional networks driving sugar metabolism reprogramming, which revealed that functional abnormalities in core glycolytic enzymes, including hexokinase 2, pyruvate kinase M2, and lactate dehydrogenase A (LDHA), are driven by a multi-level regulatory network that includes altered transcription factor activity, epigenetic modifications, and interactions mediated by non-coding RNAs. Mitochondrial dysfunction leads to succinate and reactive oxygen species accumulation, synergistically promoting the remodeling of metabolic pathways. Furthermore, the synergistic interaction between the pentose phosphate pathway and glutamine metabolism accelerates the energy supply and biosynthesis, whereas lactate accumulation shapes the immunosuppressive microenvironment by inhibiting immune cell function. This review focuses on dissecting the core mechanisms of glucose metabolism reprogramming in osteosarcoma pathogenesis, including promoting proliferation, inhibiting apoptosis, enhancing invasion and metastasis, regulating immune metabolism, and mediating chemotherapy resistance. Additionally, it evaluates novel therapeutic strategies targeting key nodes of glucose metabolism in osteosarcoma, including advances in preclinical research on specific small-molecule inhibitors and gene regulatory approaches. Finally, it delves into the major challenges in translational research, such as metabolic heterogeneity, targeting selectivity, and compensatory resistance, as well as prospects for future research, including multi-omics analysis, smart delivery systems, organoid models, and combination immunotherapy. This study offers novel insights into overcoming the therapeutic bottlenecks in osteosarcoma treatment.

## Introduction

Osteosarcoma is a highly invasive metastatic malignant bone tumor, with a poor prognosis often due to early pulmonary metastasis.^1^^,^^2^ This disease is the second most common malignant bone tumor in children and adolescents, typically occurring at the metaphysis of long bones, such as the distal femur and proximal tibia.^3^ The hallmark features of osteosarcoma manifest as pathological deposition of osteoid matrix, malignant proliferation of mesenchymal progenitor cells within the bone microenvironment, and pronounced invasive biological behavior.^2^, ^3^, ^4^ Osteosarcoma also has a significant tendency to metastasize to the lung, which is a crucial pathological feature contributing to its clinical treatment resistance and poor prognosis.^5^ Over the past several decades, treatment protocols for osteosarcoma have been continuously optimized. Particularly, neoadjuvant therapy and extensive tumor resection have improved the survival rates of patients with osteosarcoma. However, the clinical outcomes and survival rates remain unfavorable because of the early occurrence of lung metastases. This necessitates a more comprehensive understanding of this malignancy to determine optimal treatment plans.^6^

Osteosarcoma cells grow rapidly and require substantial amounts of nutrients for survival. Consequently, they undergo significant metabolic reprogramming, characterized by enhanced aerobic glycolysis, also known as the Warburg effect, which manifests as increased glucose uptake and lactate production, to meet the energy and biosynthetic demands of rapid proliferation. Despite its low energy-yielding efficiency, this process provides tumor cells with biosynthetic precursors and contributes to the maintenance of an acidic microenvironment, thereby promoting invasion, metastasis, and drug resistance.^7^, ^8^, ^9^, ^10^

Over the past few decades, research has extensively demonstrated glycolysis to be closely associated with the growth, invasion, and therapeutic response of various tumors, including osteosarcoma. Therefore, in view of the pivotal role of glucose metabolic reprogramming in the development and progression of osteosarcoma, this study aimed to systematically analyze its multidimensional regulatory network, which encompasses the molecular mechanisms of key enzymes, interactions with the microenvironment, and drug resistance. Additionally, we aimed to review the latest advances in targeted therapeutic strategies, offering new perspectives for overcoming treatment bottlenecks in osteosarcoma.

## Core mechanisms of glucose metabolism reprogramming

### Regulation and functional reprogramming of key glycolytic enzymes

Hexokinases (HK), particularly the HK2 subtype, are key enzymes that initiate glycolysis. They bind to the voltage-dependent anion channel (VDAC) and utilize adenosine triphosphate (ATP) from mitochondria to phosphorylate glucose into glucose-6-phosphate.^11^ HK2 is overexpressed in multiple malignant tumors, including osteosarcoma, where it directly mediates and enhances aerobic glycolysis in tumor cells, significantly promoting tumor growth, metastasis, and chemotherapy resistance.^11^, ^12^, ^13^ Correspondingly, targeted silencing of HK2 effectively inhibits malignant tumor progression, highlighting its central role in tumor metabolism.^14^

Abnormal HK2 overexpression is regulated by an extremely complex multilevel regulatory network. At the transcriptional level, its expression exhibits a distinct cancer-type specificity. In lung cancer models, HK2 is primarily driven by the transcription factor MYC,^15^ and hypoxia-inducible factor 1 alpha (HIF-1α) serves as a pivotal regulator of its expression.^16^ Meanwhile, in gastric cancer models, signal transducer and activator of transcription 3 (STAT3) plays a crucial synergistic regulatory role along with HK2.^17^ The activity and stability of these key transcription factors themselves are also strictly regulated, thereby indirectly amplifying the HK2 expression signal. In breast cancer, the deubiquitinating enzyme OTUB1 specifically blocks ubiquitination at the K323 site of the MYC protein, thereby delaying its degradation and enhancing its stability.^18^ In gastric cancer, lncRNA SNHG26 utilizes an internal ribosomal entry site (IRES) to activate the c-Myc–HK2 signaling axis.^19^ Notably, a synergistic effect exists between transcription factors that further finely regulate HK2 expression. For example, c-Myc and HIF-1 synergistically induce HK2 transcription and jointly promote the production of vascular endothelial growth factor (VEGF) to enhance tumor angiogenesis, tightly linking metabolic reprogramming with pro-angiogenic effects.^20^ At the epigenetic level, studies in lung adenocarcinoma models have indicated that the reduced activity of the histone methyltransferase NSD3 leads to sustained phosphorylation of STAT3 (p-STAT3Ser727), thereby driving the expression of a series of glycolytic genes, including HK2.^21^ Collectively, these findings highlight the complexity of the HK2 regulatory network and delineate the intricate nature of HK2 expression, which profoundly influences tumor metabolic adaptation and malignant progression.

Pyruvate kinase isozyme type M2 (PKM2), a key rate-limiting enzyme in glycolysis, regulates glycolytic flux through dynamic conversion between highly active tetramers and less active dimers.^22^ Disruption of this equilibrium drives malignant phenotypes in various cancers. For instance, a study utilizing colorectal cancer models shows that nuclear-localized MYG1 promotes PKM2 phosphorylation by recruiting the heat-shock protein90/glycogen synthase kinase 3 beta (HSP90/GSK3β) complex, thereby accelerating glycolysis mediated by the MYC pathway.^23^ Conversely, MYG1 inhibits functional PKM2 production by blocking its alternative splicing through the degradation of the splicing factor SRSF5, mediated by the E3 ubiquitin ligase TRIM72.^24^ In ovarian cancer models under hypoxic microenvironments, HIF-1α up-regulates endothelial cell-specific molecule 1 (ESM1), which further mediates PKM2 small ubiquitin-like modifier modification (SUMOylation) and dimerization, significantly enhancing the Warburg effect.^25^ Notably, the tetrameric state of PKM2 exhibits dual effects of tumor suppression and promotion. For example, in renal cell carcinoma, the PKM2 all-osteosarcoma-specific converter PAC responds to O-GlcNAcase, which is overexpressed in the tumor microenvironment. PAC self-assembles into nanofiber structures, continuously exposing the PKM2 serine activation site and forcing the conversion of PKM2 into highly active tetramers. This effectively inhibits glucose consumption and lactate production.^26^ Additionally, PKM2 tetramerization prevents the nuclear translocation of its dimeric form, thereby avoiding the activation of oncogenic transcription programs upon nuclear entry. Consequently, this ultimately restores tumor cell sensitivity to chemotherapeutic agents such as sunitinib.^26^ Conversely, in glioblastoma, ALDH1A3-mediated PKM2 tetramerization promotes drug resistance: ALDH1A3 directly binds to the PKM2 protein, enhancing its tetrameric state to increase pyruvate kinase activity, leading to substantial intracellular lactate accumulation.^27^ The excess lactate drives lactylation modification at the K247 site of X-ray cross-complementing protein 1 (XRCC1), altering its surface charge distribution. This enhances its affinity for the nuclear transport protein importin α, thereby driving XRCC1 nuclear translocation and accelerating DNA damage repair, eventually mediating resistance to radiotherapy and chemotherapy.^27^ In addition, the nuclear function of PKM2 plays a crucial role in osteosarcoma. lncRNA CCAT1 (LncCCAT1) promotes the dephosphorylation of PKM2 at serine 37 by binding to PKM2 and recruiting the phosphatase Cdc25A, thereby facilitating its nuclear translocation and activating the MYC glycolysis pathway. Subsequently, PKM2 within the nucleus phosphorylates sterol regulatory element-binding protein 2 (SREBP2) at threonine 610, stabilizing the SREBP2 protein and synergistically enhancing glycolysis and lipid synthesis.^28^

LDHA, a pivotal enzyme in the final step of glycolysis, catalyzes the conversion of pyruvate to lactate. LDHA overexpression in tumor cells directly triggers microenvironmental acidosis, while significantly promoting tumor invasion, angiogenesis, and immune suppression, thereby profoundly impacting malignant tumor progression.^29^, ^30^, ^31^, ^32^

The activity of LDHA and the dysregulation of the glycolytic process are influenced by a multi-level regulatory network in which non-coding RNAs play a crucial role. For example, during osteoblast differentiation, miR-34a effectively suppresses aerobic glycolysis by directly targeting the 3′ untranslated region of LDHA mRNA.^33^ In the osteosarcoma model, circular RNA 0000376 (CIRC-0000376) acts as a molecular sponge to sequester miR-577, thereby resulting in the inhibition of HK2 and LDHA. This process enhances tumor cell growth, invasion, and glycolytic activity.^34^ Meanwhile, research on breast cancer models indicates that lncRNA TMEM105 (lncTMEM105) promotes lactate production by competitively binding to miR-1208, resulting in the targeted suppression of LDHA.^35^ Moreover, in liver cancer, the lncRNA HULC, which has RNA-binding capabilities, directly interacts with LDHA and PKM2 and synergistically enhances aerobic glycolytic flux.^36^

At the epigenetic and post-translational modification levels, studies on pancreatic ductal adenocarcinoma have revealed complex regulatory circuits. Lactylated modification of histone H3 at lysine 18 drives enhanced LDHA activity by activating the threonine–tyrosine kinase/BUB1 mitotic checkpoint serine/threonine-kinase B (TTK/BUB1B-P300) positive feedback loop.^37^ Within this circuit, the spindle assembly checkpoint protein BUB1B up-regulates expression of the histone-modifying enzyme P300, promoting amplification of histone H3 lysine 18 lactylation (H3K18la) modification. Concurrently, the serine/threonine kinase TTK directly phosphorylates tyrosine 239 of LDHA, enhancing its catalytic activity and accelerating lactate production.^37^ Notably, this circuit drives the remodeling of the immunosuppressive microenvironment during liver metastasis of pancreatic ductal adenocarcinoma, characterized by CD8^+^ T cell functional exhaustion and immune escape, which is mediated by the immune checkpoint molecule Nectin-2.^38^

### Mitochondrial dysfunction and metabolic conversion

Mitochondrial dysfunction leads to impaired electron transport chain (ETC) function and reduced ATP production, forcing tumor cells to compensate for energy demands by up-regulating aerobic glycolysis. Succinate dehydrogenase (SDH) is a crucial mitochondrial enzyme complex composed of the SDHA, SDHB, SDHC, and SDHD subunits.^39^ Functional abnormalities in SDH, such as SDHB mutations, cause succinate accumulation in various tumors.^40^^,^^41^

Accumulated succinate stabilizes HIF-1α through a dual mechanism; it inhibits prolyl hydroxylase (PHD) activity, blocking prolyl hydroxylation at positions 402 and 564 of HIF-1α. Conversely, it competitively occupies the α-ketoglutarate-dependent catalytic site of PHD.^42^, ^43^, ^44^ Furthermore, copper ions prolong the half-life of HIF-1α by inhibiting its ubiquitination.^45^ Stable HIF-1α transcription activates pyruvate dehydrogenase kinase (PDK1/3) and inhibits the expression of dihydrolipoamide S-acetyltransferase (DLAT)—a key component of the pyruvate dehydrogenase complex (PDHC)—while directly inducing glycolytic genes such as GLUT1, HK2, and LDHA, thereby enhancing glycolytic flux.^42^^,^^45^ Concurrently, succinate accumulation due to SDH deficiency induces reverse electron transport, leading to highly reduced nicotinamide adenine dinucleotide (NADH). This increases electron leakage and results in excessive reactive oxygen species (ROS) production.^46^ Notably, in an influenza A virus model, excessive ROS enhanced the stability of HIF-1α by suppressing PHD activity and activating its transcription.^47^ Additionally, ROS can regulate glycolysis through multiple pathways, including NF-κB,^48^ phosphoinositide 3-kinase/protein kinase B (PI3K/AKT),^49^ Yes-associated protein (YAP),^50^ and mechanistic target of rapamycin/AMP-activated protein kinase (mTOR/AMPK).^51^

Cytochrome c oxidase (COX), also known as terminal complex IV of the ETC, primarily catalyzes the reduction of oxygen to water. Functional and structural impairment of COX leads to a decreased efficiency of oxidative phosphorylation (OXPHOS) and is accompanied by ROS accumulation.^52^^,^^53^ In mammals, this complex typically comprises 13 subunits. Among them, COX1, COX2, and COX3, encoded by mitochondrial DNA, form the core oxygen-binding subunits, while COX1 and COX2 are responsible for heme binding and copper ion assembly.^54^ The remaining subunits are encoded by nuclear DNA.^55^ Mitochondrial DNA mutations can severely impair COX function. For instance, MT–CO1 missense mutations disrupt heme A binding, MT–CO2 mutations interfere with the assembly of the copper ion center CuA, and MT–CO3 defects lead to the accumulation of immature subcomplexes.^56^ Additionally, the mitochondrial translational activator TACO1 stabilizes the peptidyl transferase center of the large ribosomal subunit, overcoming ribosomal stalling caused by polyproline sequences and accelerating the synthesis of COX1 and COX3.^57^ Mitochondrial fission process protein 1 (MTFP1) regulates mitochondrial DNA copy number and COX subunit expression by modulating inner membrane fusion dynamics.^58^ At the gene regulation level, the coactivator 1α (PGC-1α) of peroxisome proliferator-activated receptor gamma (PPARγ) binds to and enhances the transcriptional activity of the nuclear respiratory factor 1 (NRF1),^59^ thereby activating the uncoupling protein 2 (UCP2) axis mediated by miR-497 and miR-195 to regulate oxidative stress.^60^ In contrast, the transcription repressor HEY2 silences the PGC-1α promoter by recruiting histone deacetylase (HDAC1), thereby diminishing NRF1 activity.^61^ Simultaneously, nuclear factor erythroid 2-related factor 2 (NRF2) down-regulates COX-2 expression by inhibiting the nuclear factor kappa-light-chain-enhancer of activated B cells (NF-κB) pathway signaling.^62^ Furthermore, under hypoxic conditions, HIF-1 overexpression induces the transcription of the COX4-2 subunit and mitochondrial protease LON. Subsequently, LON mediates COX4-1 degradation to reconfigure complex function.^63^ While surfeit locus protein 1 (SURF1) stabilizes mature complex formation by promoting core subunit assembly, its deficiency leads to intermediate accumulation and respiratory chain collapse.^64^^,^^65^

Collectively, these findings indicate that abnormal accumulation of succinate, triggered by mitochondrial dysfunction, ROS-induced proliferation of osteosarcoma cells, and dysregulation of the COX complex constitute a core triad of mechanisms driving tumor metabolic reprogramming.

### Synergy and divergence in metabolic networks

The pentose phosphate pathway (PPP), a branch of glycolysis, generates nicotinamide adenine dinucleotide phosphate (NADPH) and 5-phosphoribose phosphate (R5P) during the oxidative phase. NADPH and R5P are utilized for oxidative stress defense and nucleotide synthesis, respectively.^66^

This pathway begins with an irreversible oxidation reaction catalyzed by glucose-6-phosphate dehydrogenase (G6PDH), yielding the first NADPH. Subsequently, 6-phosphogluconate dehydrogenase (6PGD) catalyzes secondary oxidation to produce NADPH and 5-phosphoribulose (Ru5P).^67^ NADPH protects tumor cells from oxidative damage by scavenging ROS and maintaining reduced glutathione (GSH) levels.^66^^,^^68^ Therefore, targeted intervention strategies for this pathway primarily involve two classes of inhibitors. The first category comprises G6PDH inhibitors, such as Bay 11-7069,^69^ andrographolide,^70^ and quercetin,^71^ which suppress enzymatic activity through distinct molecular mechanisms. The second category comprises 6PGD inhibitors, including physcion and its derivative S3,^72^ pomegranate peel,^73^ and gambogic acid,^74^ which have demonstrated selective inhibitory effects in lung and breast cancer models, and mouse xenograft tumors, respectively.

The non-oxidative phase of the PPP is regulated by the cooperative action of transketolase (TKT) and transaldolase (TALDO). TKT catalyzes the conversion of R5P and Xu5P into glyceraldehyde-3-phosphate (G3P) and sedoheptulose-7-phosphate (S7P).^75^ TALDO further catalyzes the formation of erythrose-4-phosphate (E4P) and fructose-6-phosphate (F6P).^66^ Research indicates that the APC/CCDH1 complex binds to TKT during the G1/S phase, dynamically regulating R5P reverse synthesis to meet DNA synthesis demands.^75^ Furthermore, F6P can re-enter the oxidation phase to replenish NADPH, while G3P is recycled back into glycolysis, forming a metabolic network switching mechanism.^66^

Glutamine, a key metabolic intermediate, plays a multidimensional role in tumor metabolic reprogramming. Glutamine maintains energy homeostasis by replenishing the tricarboxylic acid cycle (TCA) intermediates when aerobic glycolysis is activated.^76^^,^^77^ Additionally, glutamine participates in the synthesis of purine and pyrimidine nucleotides while simultaneously serving as a nitrogen and carbon source.^78^, ^79^, ^80^ Moreover, its metabolites can be further converted into precursors of glutathione, which plays an important role in regulating the cellular redox status, thereby protecting tumor cells from oxidative stress damage.^77^^,^^81^^,^^82^ Notably, glutamine provides a molecular framework for the synthesis of non-essential amino acids such as alanine and aspartic acid through transamination reactions, highlighting its pivotal position within metabolic networks.^83^^,^^84^

The abnormal proliferation of cancer cells relies on the remodeling of glutamine metabolism, which maintains the energy supply by activating oncogenes to reconstruct core pathways, such as the TCA cycle. Current research focuses on the key nodes in carcinogenic signaling that regulate metabolic networks, revealing their molecular mechanisms. The c-Myc oncogene drives glutamine addiction via a dual mechanism. While it directly up-regulates the expression of the transporter complex solute carrier family 1 member 5 (SLC1A5) and solute carrier family 1 member 7 (SLC7A5), enhancing glutamine uptake efficiency and activating mTORc1,^85^ it also accelerates glutamine catabolism by suppressing miR-23a/b to release transcriptional repression on glutaminase (GLS).^86^ A subsequent study revealed that LINC01764 synergistically promoted glucose and glutamine metabolic pathways by up-regulating c-Myc; its silencing activated uridine phosphoribosyltransferase 1 (UPP1), a key enzyme in 5-fluorouracil (5-FU) metabolism, thereby inducing 5-FU resistance.^87^ Current research on this pathway has revealed multiple intervention strategies, exemplified by the following three approaches. First, *Qici Sanling* decoction (QCSL) inhibits c-Myc expression, thereby down-regulating the key glutamine metabolism proteins GLS1 and SLC1A5. This blocks glutamine uptake and degradation, and induces apoptosis in bladder cancer cells.^88^ Second, JHU395, a prodrug derivative of l-norleucine and 6-diazo-5-oxo-l-norleucine (DON), releases an irreversible inhibitor of glutaminase activity after crossing the blood–brain barrier, thereby targeting MYC-driven glutamine dependency in medulloblastoma.^89^ Additionally, Ginsenoside Rk1 inhibits GLS1 expression and activity via the ERK/c-Myc signaling axis, triggering glutathione depletion and ROS accumulation, ultimately inducing mitochondrial apoptosis.^90^

α-Ketoglutaric acid (α-KG), as the core end product of glutamine metabolism, is synthesized from glutamine via the action of mitochondrial enzymes, including glutamic pyruvic transaminase 2 (GPT2) and glutamic-oxaloacetic transaminase 2 (GOT2).^78^ This metabolite activates the mTORC1 pathway in a PHD-dependent manner, thereby driving a complex pro-cancer effect.^91^^,^^92^ At the level of DNA damage repair, mTORC1 activation drives the pro-neural–mesenchymal transition process and up-regulates the expression of DNA repair factors through the MSN–PI3K/mTOR–CEBPB/STAT3 transcriptional cascade, thereby significantly enhancing the repair capacity of double-strand breaks mediated by γ-H2AX.^93^ At the autophagy inhibition level, mTORC1 establishes a triple blockade mechanism; phosphorylation of unc-51 like autophagy activating kinase 1 (ULK1) (Ser758) disrupts the AMPK–ULK1 interaction to inhibit autophagy initiation; phosphorylation of ATG14L inhibits VPS34 lipid kinase and block autophagosome nucleation; and trapping of transcription factors on the lysosomal surface via the GTPases Ras-related GTPases/transcription factor EB (Rag GTPases-TFEB) (Ser211) axis suppresses autophagy gene transcription.^94^ Lysosomes play a pivotal role in immune regulation. Vps33B-mediated late endosome–lysosome fusion clears the LYNUS complex (containing Rag/RHEB/mTOR), thereby limiting the excessive activation of mTORC1. When this regulation is disrupted, mTORC1 up-regulates the expression of HK2 and PDK1 in regulatory T cells, thereby enhancing CTLA-4/CD25-mediated immunosuppression. Concurrently, it inhibits receptor tyrosine kinase signaling via S6K1-IRS1/GRB10 negative feedback, thereby synergistically weakening anti-tumor immune responses.^95^

Thus, the PPP provides essential nucleotide precursors and antioxidant defense support for tumor proliferation, whereas glutamine metabolism continuously supplies biosynthetic substrates and replenishes energy. Under dominant regulation by the c-Myc transcription factor, these pathways form a metabolic synergy, jointly establishing an energy foundation for malignant growth.

### Metabolite interactions and microenvironmental dynamic regulation

LDHA-mediated lactic acid accumulation forms an immunosuppressive hub in the tumor and drives immune evasion via multiple mechanisms. For instance, accumulated lactate directly lowers intracellular pH in natural killer cells, inhibiting nuclear factor of activated T cells (NFAT) activation and interferon gamma (IFN-γ) expression while depleting ATP reserves and impairing cytotoxic function. Concurrently, LDHA synergistically suppresses anti-tumor immune responses by inducing the infiltration of myeloid-derived suppressor cells. This mechanism is significantly correlated with LDHA expression levels and survival prognosis in melanoma and pancreatic cancer models.^96^

At the molecular modification level, the alanine tRNA synthetases AARS1/2 can detect high-lactate environments and mediate the lactylation modification of key proteins. Mechanistically, under high-lactate conditions, AARS2 interacts with cyclic guanosine monophosphate-adenosine monophosphate synthase (cGAS), mediating N-terminal lactylation of the cGAS protein. This inhibits the ability of cGAS to bind to DNA, disrupts its liquid-liquid-phase-separation structure, and suppresses downstream cGAMP synthesis.^97^ Upon activation of the epidermal growth factor receptor (EGFR), ERK phosphorylation-mediated nuclear translocation of acyl-CoA synthetase short chain family member 2 (ACSS2) promotes the formation of the LDHA/ACSS2/KAT2A complex, thereby enhancing histone lactoylation modification to facilitate immune evasion.^98^ Notably, lactate secreted by PKM2 up-regulation can accelerate tumor progression by suppressing Galectin-9 transcription via NF-κB pathway inhibition.^99^

The inhibitory effect of lactate on immune cells exhibits targeted specificity. For instance, G-protein coupled receptor 132 (GPR132) has been identified as a key receptor for the perception of lactate by natural killer (NK) cells. Lactate accumulation can disrupt NK cell energy metabolism and reduce IFN-γ production by suppressing NFAT activity, thereby diminishing their cytotoxic activity.^31^^,^^95^ Furthermore, GPR132 activation via the GαS signaling pathway causes NK cell dysfunction while simultaneously inducing M2-like polarization of macrophages, which indirectly suppresses NK cell function.^100^

Lactic acid exerts potent inhibitory effects on T cells. Terminally exhausted T cells specifically express monocarboxylate transporter 11 (MCT11), which internalizes lactic acid into terminally exhausted T cells, causing intracellular lactate accumulation that inhibits OXPHOS and promotes histone lactylation, while up-regulating the expression of exhaustion genes, including programmed cell death protein 1 (PD-1) and Tim3.^101^ Moreover, lactate competitively binds to glucose transporter type 10 (GLUT10), which is highly expressed on activated CD8^+^ T cells, inhibiting key glycolytic enzymes and thereby disrupting T cell metabolism.^32^^,^^102^ Microenvironmental lactate concentrations enhance histone H3 lysine 27 (H3K27) acetylation at the Tcf7 locus by inhibiting HDAC activity and specifically up-regulating T cell factor 1 (TCF-1) expression to maintain stem cell characteristics.^103^

Notably, lactate exerts a concentration- and environment-dependent regulation of immune responses. Low pH (pH ≤ 6.4) accompanied by high lactate (≥ 15 mM) concentrations, typically exerts an immunosuppressive effect,^96^^,^^101^ whereas the lactate anion enhances glycolysis, thereby weakening the post-transcriptional suppression of IFN-γ/IL-2/TNF-α mRNA by GAPDH. This significantly boosts T cell cytokine secretion and enhances anti-tumor immune responses.^104^ Alternatively, this process specifically up-regulates TCF-1, thereby promoting T cell stemness and strengthening anti-tumor functions.^103^

In addition to lactate, metabolic and signaling molecules actively secreted by tumor cells or delivered via exosomes also play a crucial role in shaping the immunosuppressive microenvironment.^105^ For example, glioma-derived exosomes deliver miR-1246 to target macrophages, activating the STAT3 pathway while inhibiting the NF-κB pathway, thereby inducing M2 polarization.^106^ Exosomes derived from oxaliplatin-resistant colorectal cancer cells transport circRNA hsa_circ_0005963 to sensitive cells, where they enhance glycolysis by up-regulating PKM2 through miR-122 sponging, thereby reversing chemotherapy resistance.^107^ Meanwhile, prostate cancer cell-derived exosomes induce immune suppression and alter macrophage polarization by up-regulating programmed death-ligand 1 (PD-L1) and inflammatory cytokine expression while inhibiting the PI3K/AKT pathway.^108^ Additionally, intervention strategies targeting exosomes, such as the folate receptor-directed nanocomplex FD9R-GW/siIRF3, delivered GW4869 to cancer cells and tumor-associated macrophages in an orthotopic breast cancer model. This inhibited the release of PD-L1-containing tumor-derived exosomes while simultaneously silencing IRF3 to inhibit the M2-type differentiation of tumor-associated macrophages.^109^ Moreover, in colorectal cancer models, the metabolite succinate activated its receptor succinate receptor 1 (SUCNR1), triggering downstream signaling cascades. This stabilized HIF-1α, up-regulated EZH2, epigenetically silenced the cGAS–STING pathway, reduced interferon beta (IFN-β), CCL5, and CXCL10 secretion, and weakened CD8^+^ T cell chemotaxis and activation, ultimately promoting immune escape.^110^ In view of the above results, lactic acid and exosome-mediated metabolic reprogramming are key factors contributing to the formation of an immunosuppressive microenvironment. These factors exert their effects via multiple mechanisms, including interference with protein modifications, regulation of receptor signaling pathways, remodeling of epigenetic states, and attenuation of anti-tumor immune responses.

### The core mechanism of glucose metabolism reprogramming in osteosarcoma pathogenesis

Reprogramming of glucose metabolism regulates the initiation and progression of osteosarcoma through a complex network. The core mechanisms are summarized in Figure 1 and encompass five key aspects: promoting osteosarcoma proliferation, suppressing apoptosis, enhancing invasion and metastasis, modulating immune metabolism, and mediating chemotherapy resistance.

**Figure 1:**
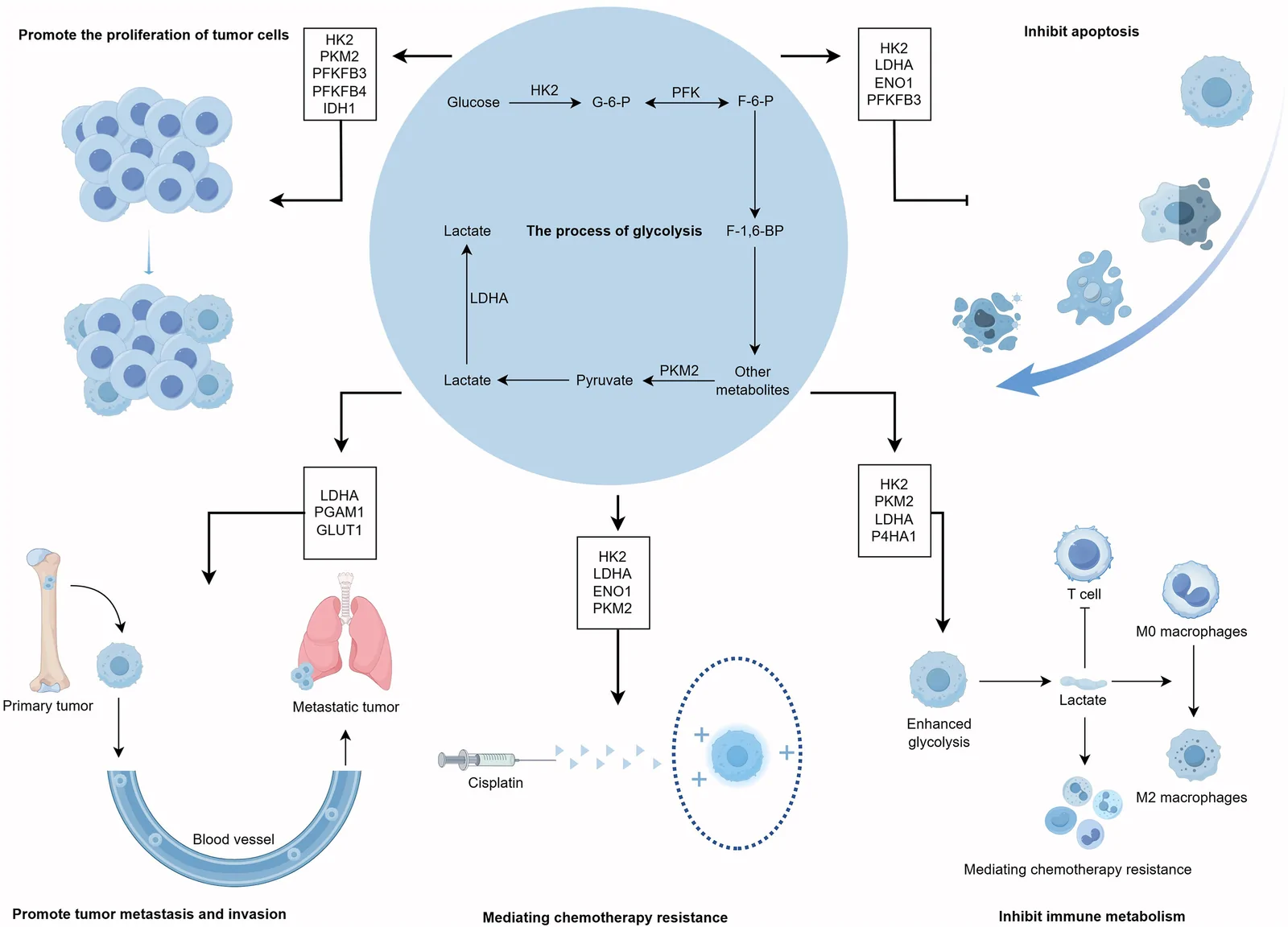
Panorama of targeted intervention strategies and molecular mechanisms of glycometabolic reprogramming in osteosarcoma.

### Promoting proliferation

In recent years, several studies have explored the role of glycometabolic reprogramming in osteosarcoma pathogenesis. P2X purinoceptor 7 (P2RX7) stabilizes c-Myc to activate glycolytic enzymes, such as HK2, GPI, and PKM2, driving the explosive production of lactate and ATP. This provides energy and microenvironmental support for osteosarcoma cells. Accordingly, targeting the P2RX7–c-Myc–metabolic axis can simultaneously inhibit energy supply and proliferation signaling.^111^ Similarly, HER4 directly activates key glycolytic enzymes, including GLUT1, HK2, and LDHA, by stabilizing c-Myc and driving comprehensive metabolic reprogramming encompassing glucose uptake, phosphorylation, and lactate production. This supplies ATP and biosynthetic precursors to osteosarcoma cells and promotes tumor growth through the lactate microenvironment.^112^ Furthermore, sine oculis homeobox 4 (SIX4) significantly enhances the transcriptional activity and expression of isocitrate dehydrogenase 1 (IDH1) by directly binding to its gene promoter. This accelerates the conversion of isocitrate to α-KG in the TCA cycle. Accumulated α-KG provides substantial ATP and NADPH to osteosarcoma cells, driving malignant proliferation.^113^

Beyond these aspects, research on N6-methyladenosine (m^6^A) modifications in tumor glycolysis has received increasing recognition in recent years. m^6^A modification is a methylation modification occurring at the N6 position of adenosine,^114^ which can regulate fatty acids,^115^ metabolic enzymes, glutamine metabolism, key components of tumor amino acid metabolism, nucleotide synthesis, and mitochondrial metabolism,^116^^,^^117^ thereby participating in tumor chemotherapy resistance and influencing lipid metabolism and chemotherapy resistance in tumor cells. In osteosarcoma tissue cells, circ-CTNNB1 directly binds to the m^6^A methyltransferase RNA binding motif protein 15 (RBM15) via its RRM1 domain, enhancing its methyltransferase activity and promoting high-density m^6^A modification in the 3′-UTR regions of key glycolytic genes such as HK2, GPI, and phosphoglycerate kinase 1 (PGK1). This modification significantly up-regulates target gene expression by prolonging the mRNA half-life and enhancing translation efficiency.^118^ These results reveal that m^6^A RNA methylation modification, as an emerging layer of post-transcriptional regulation, plays a crucial role in the glycometabolic reprogramming and progression of osteosarcoma. Consequently, this provides new insights for future osteosarcoma therapies and opens new avenues for the development of specific inhibitors targeting osteosarcoma glycolysis.

Additionally, sphingosine 1-phosphate receptor 3 (S1PR3) and its ligand S1P are highly expressed in osteosarcomas. Their signaling axis promotes YAP nuclear translocation by inhibiting YAP phosphorylation, forming a YAP–c-Myc complex that directly activates glycolytic enzymes such as phosphoglycerate mutase 1 (PGAM1). However, the combination of S1PR3 antagonists with methotrexate effectively suppresses this proliferative effect.^119^ Abnormal expression of keratin 17 (KRT17) has been reported in multiple cancers; therefore, Yan et al investigated their roles in osteosarcoma. Quantitative reverse transcription PCR and Western blotting results indicated that KRT17 was closely associated with the AKT/mTOR/HIF-1α pathway. Knockdown of KRT17 induced cell cycle arrest at the G1 phase and inhibited key indicators of glycolysis, such as the extracellular acidification rate (ECAR), cellular oxygen consumption rate (OCR), and ATP uptake, as well as the expression of downstream molecules, including VEGF, GLUT1, and MCL1.^120^ Furthermore, S100 calcium-binding protein A10 (S100A10) has also been found to activate the AKT/mTOR pathway and promote the malignant characteristics and glycolysis of osteosarcoma cells. The AKT activator SC-79 reversed this tumor-promoting effect of S100A10.^121^

In contrast, Caudatin, a C-21 steroidal glycoside extracted from the root of *Cynanchum bungei* Decne, has been reported to exhibit anti-cancer activity.^122^ Caudatin inhibits the proliferation and glycolytic capacity of osteosarcoma cells by suppressing the Wnt/β-Catenin pathway.^123^ Additionally, Rho-associated coiled-coil kinase 2 (ROCK2) promotes HK2 expression by activating the phospho-PI3K/AKT pathway.^124^ Lysine (K)-specific demethylase 3A (KDM3A) synergistically regulates SP1 to up-regulate the expression of 6-phosphofructo-2-kinase/fructose-1,6-isphosphatase 4 (PFKFB4), thereby promoting osteosarcoma cell proliferation and glycolytic capacity.^125^

At the post-translational modification level, deubiquitinating enzymes play a crucial role in stabilizing metabolic enzymes or their upstream regulatory factors. Ubiquitin-specific protease 33 (USP33) stabilizes glycolytic rate-limiting enzyme 6 via deubiquitination, while phosphofructo-2-kinase/fructo-osteosarcoma-2,6-biphosphatase 3 (PFKFB3) drives osteosarcoma proliferation and glycolytic capacity.^126^ Similarly, ubiquitin-specific protease 22 (USP22) stabilizes β-catenin, thereby up-regulating HK2 expression.^127^ Conversely, down-regulation of USP22 significantly reduces the expression levels of other core glycolytic enzymes, including PKM2 and GLUT1, in SAO-2 cells.^128^ Therefore, deubiquitinating enzymes like USP22 and USP33 may serve as indispensable molecular mechanisms for maintaining high glycolytic activity and sustained proliferation in osteosarcoma by stabilizing glycolytic enzymes or their upstream regulators.

### Inhibition of apoptosis

Early studies have demonstrated that cytochrome c is released into the cytoplasm during apoptosis and promotes the activation of caspases, an indispensable family of proteases in the apoptotic process.^129^^,^^130^ Subsequently, a direct link between glucose metabolism and this apoptotic pathway was uncovered. The mitochondrial outer membrane protein, VDAC, interacts with Bax to form channels for cytochrome c passage. Notably, Pastorino et al have demonstrated that HK2 competitively binds to VDAC and localizes to the mitochondria, thereby inhibiting cytochrome c release and preventing apoptosis.^131^

Myc regulates multiple key downstream glycolytic genes.^132^ Recent studies have indicated that P2RX7 modulates the activity of key downstream enzymes by stabilizing c-Myc metabolic reprogramming, ultimately inhibiting apoptosis in osteosarcoma cells.^111^ Similarly, the mTOR pathway is highly activated in clinical osteosarcoma samples.^51^ Further investigation by Wang et al on the mTOR pathway indicated that the mTORC1/serine/glycine metabolic axis enhances the antioxidant capacity of osteosarcoma cells, thereby inhibiting apoptosis.^133^

In addition to canonical pathways, other regulators influence apoptosis through glycolysis. For instance, COP9 influences apoptosis and regulates glycolysis.^134^ Zhang et al first reported that COP9 signalosome subunit 3 (COPS3) was highly expressed in osteosarcoma cells and functioned as an upstream regulator of PFKFB3, thereby inhibiting cell apoptosis via the glycolysis pathway.^135^ This finding suggests the multidimensional regulatory properties of the mTOR network.

Genetic alterations are another layer of regulation. Gene fusion is a common phenomenon in tumors and plays a crucial role in promoting tumorigenesis and progression.^136^ Clathrin heavy chain (CLTC) and vacuole membrane protein 1 (VMP1) are two genes associated with intracellular substance transport and phagosome formation.^137^ Zou et al have revealed that the CLTC–VMP1 fusion gene, formed by the fusion of the 14th exon of CLTC with the last two exons of VMP1, functions as a key molecular switch in regulating the balance between glucose metabolic reprogramming and apoptosis.^138^ However, its underlying regulatory mechanisms remain unclear.

Finally, at the post-translational level, deubiquitination plays a crucial role in suppressing apoptosis of osteosarcoma cells. For instance, the deubiquitinating enzyme USP13 stabilizes methyltransferase-like 3 (METTL3) to regulate autophagy-related 5 (ATG5) mRNA, thereby maintaining the autophagy-apoptosis balance.^139^ This highlights the complexity of the interactions between epigenetic modifications and metabolism. The discovery of the CLTC–VMP1 fusion gene and deubiquitinating enzymes such as USP13 has revealed novel mechanisms linking genetic abnormalities and post-translational modifications in the regulation of metabolic reprogramming and apoptosis.

### Enhanced invasion and metastasis

Recent research has revealed multilevel regulatory mechanisms underlying the invasion and metastasis of osteosarcoma, with epigenetic modifications and metabolic reprogramming playing pivotal roles in these processes. For instance, Jiang et al found that lysine demethylase 6B (KDM6B) directly activated the transcription of LDHA by removing histone H3 lysine 27 trimethylation (H3K27me3) modifications, thereby promoting glycolysis and driving metastatic phenotypes. Notably, KDM6B knockout significantly suppressed osteosarcoma cell migration and lung metastasis *in vivo*, whereas LDHA overexpression reversed this effect, suggesting that the KDM6B–LDHA axis is a potential therapeutic target.^140^ Additionally, Shi et al revealed that leucine-containing zipper and EF-hand transmembrane protein 1 (LETM1) promoted metastasis through a dual mechanism. LETM1 enhanced self-renewal capacity by up-regulating Nanog/SOX2/SOX9 to maintain tumor stem cell properties and regulated cell cycle dynamics by reducing S-phase arrest, thereby boosting proliferative potential. Accordingly, LETM1 knockdown significantly inhibited wound healing and Transwell migration abilities, while reducing the formation of lung metastatic lesions *in vivo*.^141^

### Regulating immune metabolism

Aerobic glycolysis in osteosarcoma cells promotes tumor invasion and metastasis through interactions between metabolites and the immune system. Genes, such as ST3GAL4, TRAM2, and P4HA1, along with the PI3K/AKT/mTOR signaling pathway, play key regulatory roles in this process.

Notably, the substantial amount of lactate produced by the Warburg effect can drive tumor-associated macrophages toward M2 polarization via the ST3GAL4/Siglec-7/9 axis. Specifically, high ST3GAL4 expression is positively correlated with elevated ECAR and increased lactate production.^142^, ^143^, ^144^ Using coculture experiments, Wu et al demonstrated that ST3GAL4 up-regulated the expression of CD206, an M2 macrophage marker. Moreover, inhibition of ST3GAL4 simultaneously reduced tumor cell glycolysis and macrophage PD-L1 expression, suggesting a bidirectional regulatory mechanism between glucose metabolism and immune suppression.^145^

Furthermore, the metabolic reprogramming of tumor cells activates the MYC and mTOR signaling pathways, forming a cycle that sustains their malignant characteristics. This manifests as the maintenance of a high proliferative capacity in tumor cells and the suppression of CD8^+^ T-cell effector function.^146^^,^^147^ Notably, this type of metabolism-mediated T-cell functional exhaustion synergizes with the PD-1/PD-L1 signaling pathway. As a key immune checkpoint, PD-1/PD-L1 can be directly exploited by tumor cells to evade immune surveillance.^148^ Additionally, tumor cell glucose metabolism levels are positively correlated with the expression of the immune checkpoint molecule HAVCR2, suggesting that metabolic reprogramming may influence T-cell function by regulating immune checkpoint molecules.^145^^,^^149^

Subsequently, Gui et al highlighted that elevated expression of the key glycolytic gene P4HA1 enhanced glycolytic activity and mediated immune microenvironment remodeling by altering the extracellular matrix collagen structure. This manifests as a reduction in CD8^+^ T cells, accompanied by increased numbers of naive CD4^+^ T cells, resting dendritic cells, and activated mast cells. Gene set enrichment analysis further confirmed that the glycolysis-high-risk group exhibited significant enrichment in pathways including adaptive immune response, antigen presentation, and complement activation compared with the low-risk group.^150^ Xu et al detected glycogenolysis and cholesterol synthesis-related gene signatures through comprehensive analysis of the GSE21257 and TARGET-OSTEOSARCOMA databases. They observed significantly increased macrophage and NK T cell infiltration in glycolysis and cholesterol synthesis-related genes (GCSRGs) with low expression, along with the up-regulation of immune checkpoint molecules (*e.g.*, LGALS9, HAVCR2, PDCD1LG2, and ICOS). Furthermore, high expression of the TRAM2 gene, via regulation of the PI3K/AKT/mTOR signaling pathway, promotes glycolysis and epithelial–mesenchymal transition in osteosarcoma cells, while simultaneously suppressing the anti-tumor activity of CD8^+^ T cells in the tumor microenvironment and promoting the infiltration of regulatory T cells.^151^

Finally, single-cell transcriptomic analysis provided further insights into the significant heterogeneity of lactate metabolism within the osteosarcoma microenvironment, where NDUFAF6 was identified as a key regulator of immune metabolism.^152^ Therefore, future research should utilize multi-omics technologies to decipher metabolic heterogeneity within the microenvironment and delve deeper into the synergistic therapeutic mechanisms of targeting glycolysis and immune checkpoint inhibitors.

### Mediated chemotherapy resistance

Resistance to chemotherapy remains a primary obstacle limiting the treatment of osteosarcoma, particularly when accompanied by lung metastases, leading to a significant decline in the five-year survival rate to approximately 20%.^153^ Although progress has been made in the field of osteosarcoma chemotherapy resistance research, studies on the role of glucose metabolism reprogramming in this context remain relatively scarce. Li et al found that CXCR4 was significantly overexpressed in drug-resistant osteosarcoma cells. CXCR4 promoted methylation at the H3R17 site by up-regulating the histone arginine methyltransferase CARM1, thereby activating YAP and inhibiting the Hippo pathway. Activated YAP directly up-regulated key glycolytic enzymes, including HK2, PKM2, and LDHA, ultimately conferring doxorubicin resistance, thus providing a multilayered regulatory mechanism for glucose metabolism reprogramming.^154^ Additionally, global alterations in energy metabolism patterns have been demonstrated to contribute to the development of drug resistance. Research indicates that the CLTC–VMP1 fusion gene reshapes cellular homeostasis through a dual mechanism (as described before). This metabolic plasticity enables drug-resistant cells to adjust their energy supply strategies dynamically, ultimately promoting the development of cisplatin resistance.^138^

In addition to glycolysis, other metabolic pathways contribute synergistically to drug resistance. For instance, lactic acid accumulation enhances DNA damage repair capacity and promotes an immunosuppressive microenvironment by inducing lactylation modifications of histone H3K9la, H3K18la, and non-histones NBS1 and TFEB, thereby synergistically driving chemotherapy resistance.^155^ Conversely, lipid metabolism reprogramming enhances DNA repair capacity and promotes the formation of an immunosuppressive microenvironment through CD36-mediated fatty acid uptake and the SREBP/FASN signaling axis, further synergistically driving chemotherapy resistance.^156^ Notably, targeted interventions in the metabolic and immune microenvironment have been shown to reverse drug resistance. For example, herbal monomers, such as quercetin, enhance the sensitivity of osteosarcoma cells to cisplatin by down-regulating PD-L1 expression and inhibiting STAT3 phosphorylation, thereby reshaping the immunosuppressive microenvironment.^157^

In conclusion, we systematically revealed the multidimensional regulatory mechanisms of glucose metabolism reprogramming in osteosarcoma chemotherapy resistance, providing crucial theoretical support for the development of combined therapeutic strategies targeting metabolic interventions.

### Core regulatory role of non-coding RNAs in glycolytic reprogramming of osteosarcoma

Non-coding RNAs constitute the central regulatory layer governing glycolytic reprogramming in osteosarcoma, with microRNAs (miRNAs) representing a particularly prominent component. A review by Rafat et al indicated that mIR-132-3p expression levels were significantly reduced in multiple tumors, including osteosarcoma, and that its mechanism of action may be associated with various pathways, such as oncogenes, signaling pathways, and glycolysis.^158^ A recurring functional pattern is that miRNAs typically suppress their downstream targets. This is exemplified by Weng et al, who identified a core mechanism through which the miR-324-3p/PGAM1 axis drives osteosarcoma invasion and metastasis by regulating glycolysis.^159^ They have demonstrated that miR-324-3p, which is markedly down-regulated in osteosarcoma, directly targets the 3′-UTR of PGAM1 to suppress its expression. This suppression significantly inhibits cell migration and invasion *in vitro* and reduces tumor volume and metastasis *in vivo*, which is reversed by PGAM1 re-expression. This mechanism aligns with other established miRNA functions in the network. For instance, MALAT1 functions as a molecular sponge for miR-485-3p, leading to direct inhibition of the c-Met and AKT/mTOR signaling pathways, which consequently suppress osteosarcoma proliferation and metastasis.^160^ Similarly, CIRC_0000376 sequesters miR-432-5p to up-regulate Bcl2 expression, thereby exerting similar oncogenic effects.^161^ Notably, LDHA regulation also plays a crucial role in osteosarcoma. Studies have revealed that in cisplatin-resistant SaOS-2 cells with elevated glucose metabolism, miR-329-3p expression is significantly down-regulated, and its overexpression markedly inhibits glucose metabolism. Bioinformatic analysis and luciferase assays confirmed that miR-329-3p directly targets LDHA in osteosarcoma cells. Furthermore, restoring LDHA expression reverses the cisplatin-sensitizing effect induced by miR-329-3p overexpression.^162^

In addition to these examples, other miRNAs such as miRNA-1297,^163^ miRNA-214-3 p,^164^ miR-564,^165^ and MiR-323a-3p,^166^ miR-141-3p,^167^ and miR-1207-5p,^168^ have also been implicated in this extensive regulatory network, further underscoring the critical role of miRNA-mediated regulation in osteosarcoma glycolytic reprogramming (please refer to Table 1 for more details).

**Table 1:** Preclinical intervention strategies targeting glycometabolism in osteosarcoma and their molecular mechanisms.

Type	Name	Targets	Regulatory direction	Functional impact	Reference
miRNA	miR-324-3p	PGAM1	Inhibit	Inhibit lactate formation	^159^
	miR-485-3p	c-MET, AKT3	Inhibit	Inhibit glycolysis, proliferation, and metastasis; enhance cisplatin sensitivity.	^160^
	miR-432-5p	BCL2	Inhibit	Promote apoptosis; inhibit proliferation	^161^
	miR-329-3p	LDHA	Inhibit	Inhibit lactate formation	^162^
	MiR-564	Akt	Inhibit	Inhibit lactate and glucose production	^165^
	miR-141-3p	GLS	Inhibit	Inhibiting glutamine metabolism enhances cisplatin sensitivity	^167^
	miR-1207-5p	G6PD	Inhibit	Inhibit NADPH production	^168^
lncRNA	CCAT1	PKM2	Promote	Recruitment of Cdc25A facilitates PKM2 dephosphorylation and nuclear translocation, thereby activating MYC transcription	^28^
	MALAT1	c-Met/AKT/mTOR	Promote	Adsorption of miR-485-3p activates pathways, promoting glycolytic enzyme expression	^160^
	XLOC-005950	PFKM	Promote	Inhibiting miR-542-3p releases its suppression of PFKM, thereby enhancing glycolytic flux	^169^
	DLGAP1-AS2	HK2	Promote	Inhibition of miR-451a lifts its suppression of HK2	^170^
	HOTAIRM1	Rheb/mTOR	Promote	Binding to miR-664b-3p activates mTOR and inhibits apoptosis	^172^
	KCNQ1OT1	ALDOA	Promote	Binding to miR-34c-5p up-regulates aldolase A, accelerating glycolysis	^175^
circRNA	Circ-0000376	HK2/LDHA	Promote	Competitive binding of miR-577 up-regulates HK2/LDHA, promoting glycolysis and invasion	^34^^,^^161^
	Circ-CTNNB1	RBM15/m^6^A	Promote	RBM15 mediates m^6^A modification to stabilize HK2/PKM2 mRNA, enhancing glycolysis	^118^
	Circ-0056285	KCNH1	Promote	Enhancing ion channel activity through the miR-1225-3p/KCNH1 axis promotes proliferation and invasion	^173^
	circATP2A2	MYH9	Promote	By adsorbing miR-335-5p to release inhibition of MYH9, it enhances osteosarcoma proliferation, glycolysis, and metastasis	^176^
	Circ-0136666	ZEB2	Promote	As a miR-593-3p molecular sponge, it activates ZEB2, accelerating osteosarcoma progression and metastasis	^177^
	Has-circ-0000566	LDHA	Promote	Sponge adsorption of miR-409-3p relieves LDHA inhibition, promoting aerobic glycolysis	^178^
	Circ-0004674	TCF4	Promote	Enhances the Wnt signaling pathway through the miR-140-3p/TCF4 axis, driving glycolysis and proliferation	^179^
	Circ-0016347	TRIM44	Promote	Activation of the PI3K/AKT pathway via the miR-1244/TRIM44 axis enhances proliferation and glycolysis	^180^
	Circ-CYP51A1	KLF12	Promote	Under hypoxic conditions, the miR-490-3p/KLF12 axis promotes migration and glycolysis	^181^
	Circ-CDK14	E2F2	Inhibit	Blocking the cell cycle via the miR-198/E2F2 axis inhibits proliferation and migration	^182^
	Circ-ATRNL1	LDHA	Promote	As a sponge for miR-409-3p, it up-regulates LDHA, driving aerobic glycolysis	^183^
	Circ-PVT1	Wnt5a/Ror2	Promote	Promotes metastasis and glycolysis by activating the Wnt5a/Ror2 signaling pathway through miR-423-5p	^184^
	Circ-CNST	LDHA/PDK1	Promote	As a sponge for miR-578, it relieves LDHA/PDK1 inhibition and enhances glycolysis	^185^
	Circ-0001721	HOXB2	Promote	Activate the epithelial–mesenchymal transition process through the miR-1200/HOXB2 axis, promoting metastasis.	^186^

lncRNAs are involved in metabolic reprogramming in osteosarcoma. For instance, Jia et al demonstrated that CRISPR/Cas9-mediated knockout of lncRNA XLOC-005950 resulted in the up-regulation of hsa-MirR-542-3p, which in turn down-regulated the expression of phosphofructokinase-morphofructokinase (PFKM).^169^ Additionally, Zheng et al elucidated the role of another lncRNA, DLGAP1-AS2, in a mouse xenograft model. They discovered that DLGAP1-AS2 overexpression down-regulated HK2 expression, leading to the suppression of proliferation and aerobic glycolysis in osteosarcoma cells. Subsequent rescue experiments confirmed that this occurred due to DLGAP1-AS2 acting as a molecular sponge for miR-451a, which ultimately up-regulated HK2 expression.^170^ Notably, the prometastatic role of this lncRNA was underscored in a tail vein injection model, wherein its silencing reduced lung metastasis by approximately 80%, revealing the multidimensional nature of this regulatory network. Furthermore, Wei et al revealed that LINC00520 blocked FBXW7-mediated enolase 1 (ENO1) ubiquitination and proteasomal degradation by binding to ENO1, thereby up-regulating ENO1 protein expression, enhancing glycolytic flux, and conferring cisplatin resistance in osteosarcoma.^171^

In addition to metabolic regulation, this regulatory layer also suppresses apoptosis. Yu et al have discovered that the lncRNA HOTAIRM1 is abnormally up-regulated in osteosarcoma cells and that its inhibition promotes apoptosis. Mechanistically, HOTAIRM1 acts as a competitive endogenous RNA (ceRNA) for miR-664b-3p, thereby elevating the expression of Rheb and subsequently activating the anti-apoptotic mTOR pathway.^172^ Similarly, Huo et al found that CIRC_0056285 suppressed apoptosis by sponging miR-1244 to up-regulate TRIM44 expression.^173^ Moreover, numerous other lncRNAs, including lncRNA FEZR1-AS1,^174^ and lncRNA KCNQ1OT1,^175^ have been demonstrated to participate in this extensive regulatory network (see Table 1 for more details). These findings systematically map specific regulatory axes, such as the DLGAP1-AS2/miR-451a/HK2 and HOTAIRM1/miR-664b-3p/Rheb/mTOR axes, which equivalently regulate glycolysis, apoptosis, and metastasis, revealing synergistic targets for therapeutic intervention in osteosarcoma.

Additionally, circular RNAs (circRNAs) play diverse regulatory roles in osteosarcoma progression, particularly by modulating glycolytic metabolism and malignant behavior. For example, circATP2A2 binds to miR-335-5p to up-regulate MYH9, promoting glycolysis and proliferation via the key glycolytic enzymes HK2 and PKM2, which is reversible upon inhibition of miR-335-5p or overexpression of MYH9.^176^ Similarly, circ-0136666 up-regulates ZEB2 by sponging miR-1244, thereby enhancing glycolysis and proliferation through HK2 and PKM2.^177^

Notably, Shen et al have revealed that hypoxia-induced Has-circ-0000566 forms a positive feedback loop with HIF-1α. HIF-1α transcriptionally activates this circRNA, which in turn stabilizes the HIF-1α protein by inhibiting VHL-mediated ubiquitination and degradation, leading to sustained expression of glycolytic genes including LDHA and GLUT1. Knockout of this circRNA reduced 143 B cell migration by 50% and decreased pulmonary metastasis by 70% in a tail vein injection model.^178^ Yang et al demonstrated another significant mechanism; circ-CTNNB1 is highly expressed in osteosarcoma and directly interacts with the nuclear m^6^A methyltransferase RBM15 via its RRM1 domain, facilitating m^6^A modification of mRNAs of glycolytic enzymes such as HK2, GPI, and PGK1 to enhance their stability. Knockdown of circ-CTNNB1 suppressed invasive phenotypes *in vitro* and lung metastasis *in vivo*, whereas RBM15 knockdown abolished the pro-invasive effect of circ-CTNNB1 overexpression. Notably, the glycolysis inhibitor 2-DG blocked circ-CTNNB1-driven invasion, confirming its dependence on glycolytic activation.^118^

Furthermore, multiple studies have identified additional circRNAs involved in this regulatory network, including circ-0004674,^179^ circ-0016347,^180^ circ-CYP51A1,^181^ circ-CDK14,^182^ circ-ATRNL1,^183^ circ-PVT1,^184^ circ-0000376,^34^^,^^161^ circ-CNST,^185^ circ-0001721,^186^ etc. These circRNAs participate in the regulation of glycolysis and proliferation in osteosarcoma through distinct molecular mechanisms (please refer to Table 1 for more details).

Collectively, these findings underscore the significance of circRNAs in the regulation of glycolytic pathways and metastatic processes in osteosarcoma through multiple molecular mechanisms, including miRNA sponging, protein complex formation, and epigenetic RNA modifications.

### Precision therapeutic strategies targeting glucose metabolism

The mechanisms of glucose metabolism reprogramming in osteosarcoma are complex and involve multiple pathways and key targets, providing abundant potential targets for developing precision therapeutic strategies. Currently, various compounds targeting different stages of glucose metabolism and related regulatory nodes are at the forefront of research; their mechanisms of action and target profiles are summarized in Figure 2.

**Figure 2:**
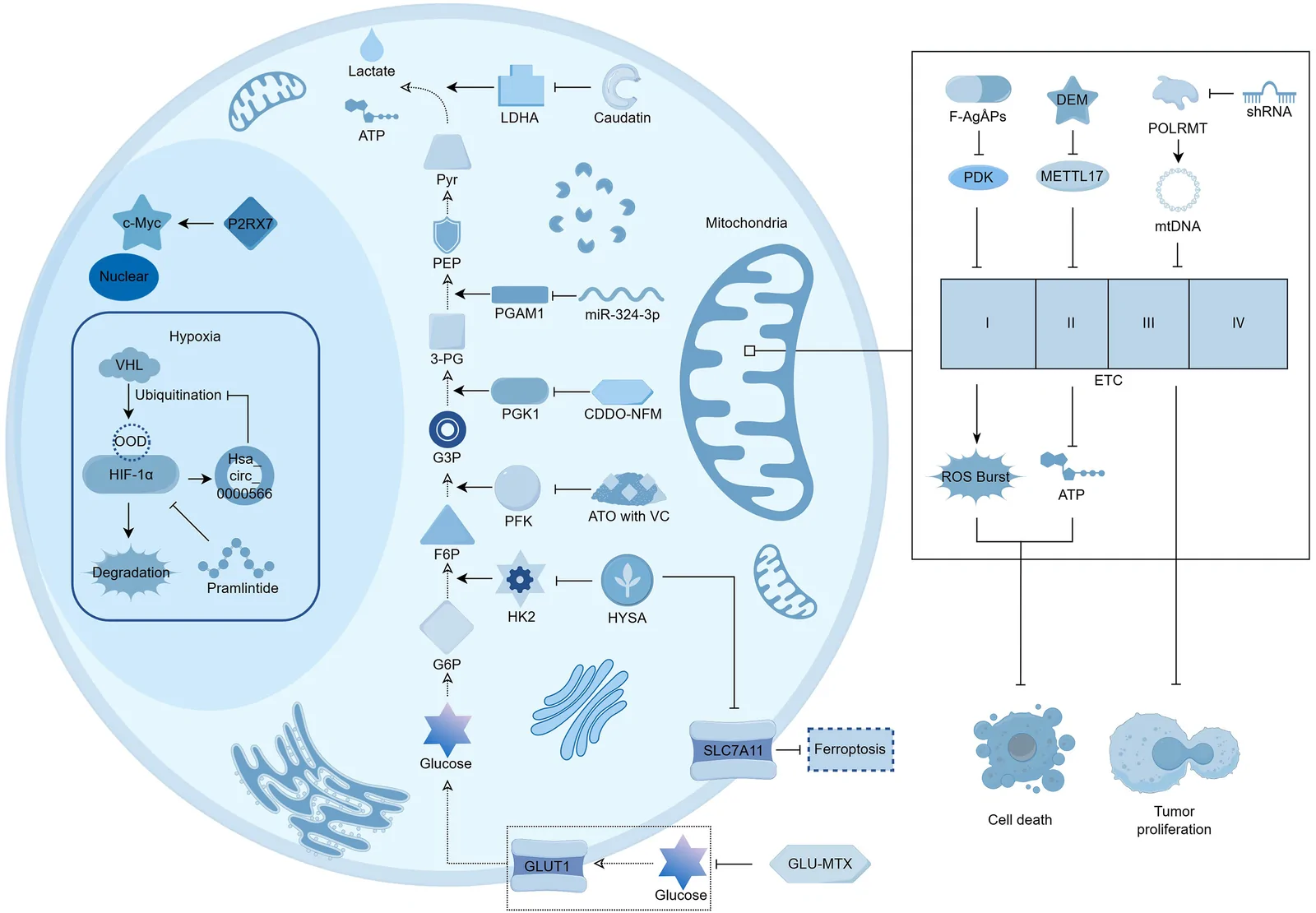
Multidimensional regulatory networks and targeted intervention strategies for glycometabolic reprogramming driving osteosarcoma.

### Inhibition of glycolysis

Precise therapeutic strategies targeting glucose metabolism have become a key direction in osteosarcoma research. Zhu et al found that hydroxysafflor yellow A (HYSA) simultaneously blocked the Warburg effect and ferroptosis (FPT) by inhibiting the dual pathways of HIF-1α/HK2 and SLC7A11. However, suppression of FPT weakened its anti-cancer activity, suggesting complex interactions between metabolic regulation and cell death pathways.^187^ Additionally, in a p53-deficient osteosarcoma model, Yang et al demonstrated that pramlintide significantly suppressed glycolytic activity by reducing HIF-1α levels while up-regulating islet amyloid polypeptide (IAPP) expression and induced tumor regression in a subcutaneous model.^188^ Moreover, methotrexate (MTX), a commonly used antimetabolite, inhibits folate and DNA synthesis. Woźniak designed a glucose-methotrexate conjugate (GLU–MTX) to address the limitations of tumor-selective targeting by MTX. In osteosarcoma cells, the uptake of GLU–MTX was 17-fold higher than that of unconjugated MTX, which significantly inhibited osteosarcoma cell growth. The inhibitory effect of GLU–MTX was partially reversed by the addition of 4,6-O-ethylidene-α-d-glucose (EDG), a GLUT1 inhibitor, suggesting that this process is mediated by glycolysis. However, the specific mechanism remains to be explored further.^189^ Natural compounds such as solasonine (SS), a naturally occurring glycoside alkaloid, also reportedly exhibit significant anti-cancer effects. Wang et al demonstrated that SS regulated glycolysis in osteosarcoma through the Wnt/β-Catenin/Snail pathway, thereby inhibiting osteosarcoma stemness and metastasis.^190^ Similarly, Zhang et al demonstrated that Caudatin, a steroidal glycoside, inhibited glycolysis in osteosarcoma cells in xenograft models via the Wnt/β-Catenin signaling pathway, highlighting the potential of this pathway for metabolic intervention.^123^

At the gene regulation level, multiple studies have revealed key molecular mechanisms; under hypoxic conditions, HIF-1α is activated and can directly bind to the hypoxia response element (HRE) within the promoter region of Hsa_circ_0000566, which is formed by the splicing of host genes. This binding significantly up-regulates the expression of this circRNA. The up-regulated Hsa_circ_0000566 specifically binds to the oxygen-dependent degradation domain (ODD) of the HIF-1α protein and the von Hippel-Lindau protein (VHL), thereby disrupting the normal interaction between VHL and the HIF-1α ODD region. Consequently, this process inhibits the degradation of HIF-1α via the ubiquitin-proteasome pathway, thereby prolonging its stability and enhancing its transcriptional activity. Thus, a positive feedback regulatory mechanism driven by HIF-1α/Hsa_circ_0000566 is established. This mechanism provides novel strategies and a theoretical basis for targeting the Hsa_circ_0000566-HIF-1α signaling axis in the treatment of osteosarcoma.^178^ CIRC_0004674 regulates glycolysis-related proteins through the miR-140-3p/TCF4 axis. Accordingly, inhibiting this gene reduces glucose consumption and lactate production.^179^ Weng et al demonstrated that miR-324-3p suppressed PGAM1 expression by targeting its 3′UTR, while elevated PGAM1 expression promoted osteosarcoma progression and aerobic glycolysis.^159^ Notably, Han et al have shown that overexpression of the cancer biomarker solute carrier family 9 member A2 (SLC9A2) can inhibit aerobic glycolysis in osteosarcoma cells while simultaneously suppressing osteosarcoma proliferation and invasion. ETS proto-oncogene 1 (ETS1) acts as a transcriptional repressor of SLC9A2. While its overexpression promotes methylation of the SLC9A2 promoter region, thereby inhibiting its transcription, inhibition of ETS1 up-regulates SLC9A2 expression, resulting in suppressed aerobic glycolysis in osteosarcoma cells.^191^ Moreover, Gao et al have shown that the N-formylmorpholine substituent of CDDO (CDDO-NFM) reduces glucose uptake, lactate production, and ATP synthesis during glycolysis in osteosarcoma cells, thereby effectively inhibiting osteosarcoma cell growth. This mechanism may be related to the degradation of c-myc.^192^ Additional in-depth mechanistic studies have revealed that P2RX7, which acts as an oncogene, enhances c-Myc stability by promoting nuclear retention and reducing ubiquitin-dependent degradation. This finding suggests that P2RX7 may serve as a potential therapeutic target for osteosarcoma.^111^ The two studies collectively highlight the central role of c-Myc in metabolic regulation. Although separate reports indicate that Arsenic trioxide (ATO) plays a significant role in the treatment of osteosarcoma, its clinical application is severely restricted due to its cardiac and hepatic toxicity. Addressing the limitations of traditional drugs, Liu et al proposed an innovative combination strategy: high-dose vitamin C (800 μM), which enhances the cytotoxicity of ATO by up-regulating Bax/Caspase-3, while synergistically inhibiting key glycolytic enzymes such as PFK, PGK1, and LDHA. This discovery offers a novel approach for overcoming the cardiac and hepatic toxicities of ATO.^193^

Collectively, these studies provide a theoretical basis for developing treatment strategies for osteosarcoma that target the cross-regulation between glycometabolism and apoptosis. Informing the clinical application of these strategies, deep-learning-based multimodal data fusion technology integrates imaging, omics, and clinical information, offering new intelligent support tools for optimizing precision decision-making in the aforementioned targeted therapies.^194^

### Interfering with oxidative phosphorylation and mitochondrial function

Fructose-coated AgÅPs (F-AgÅPs) developed by Hu et al (particle size 9.38 ± 4.11 nm) demonstrated exceptional stability *in vivo* and *in vitro*. In an osteosarcoma nude mouse model, intravenous injection of F-AgÅPs significantly inhibited tumor growth with superior efficacy compared with cisplatin, while improving survival rates and reducing bone resorption and lung metastasis. The mechanism likely involves the inhibition of PDK by F-AgPs, thereby selectively shifting osteosarcoma cells from glycolysis to oxidative phosphorylation (OXPHOS), ultimately inducing ROS-dependent apoptosis in these cells.^195^ Similarly, Cao et al discovered that demethylzeylasteral (DEM) impaired OXPHOS by targeting the mitochondrial translational regulator methyltransferase-like 17 (METTL17), resulting in ATP depletion. METTL17 knockout inhibited osteosarcoma growth, whereas its overexpression reversed the effects of DEM and restored ATP production.^196^

Additionally, multiple studies have revealed a central role for ROS. Vitamin C exhibits pro-oxidative properties that selectively induce cancer cell death. Vaishampayan et al have demonstrated that vitamin C mediates ROS-iron-calcium crosstalk in osteosarcoma cells and induces mitochondrial dysfunction through its redox activity. This mechanism likely involves triggering calcium release via inositol 1,4,5-trisphosphate receptors (IP3Rs) by vitamin C, leading to mitochondrial ROS production and ultimately cell death.^197^ Yao et al illustrated that manoalide (a phospholipase A2 inhibitor with anti-inflammatory properties) could inhibit MG 63 cell activity by 45.13%–4.40%. Concurrently, another study reported that manoalide induced excessive ROS production, disrupting antioxidant proteins and activating apoptotic proteins caspase-9/-3 and PARP (Poly (ADP-ribose) polymerase), ultimately causing mitochondrial dysfunction and apoptosis in osteosarcoma cells.^198^ Quigley et al revealed that 1,25-dihydroxyvitamin D, 1,25(OH) (2)D (vitamin D_3_), reduced mitochondrial ROS levels, membrane potential, and biosynthetic/translational activity in the vitamin D receptor (VDR)-sensitive MG-63 osteosarcoma cell model. Subsequent gene enrichment pathway analysis indicated that 1,25(OH)_2_D down-regulated the mitochondrial fusion/fission and OXPHOS pathways. The mechanism may involve the cytoplasmic translocation of the oxidative stress sensor DDIT4/REDD1, which promotes metabolic reprogramming toward non-oxidative pathways by inhibiting mTORC1 signaling, thereby synergistically restoring redox homeostasis and suppressing tumor proliferation.^199^

Meanwhile, Li et al uncovered key insights into the antler growth mechanisms in deer. Inner cells within the reserve mesenchyme exhibit high proliferative characteristics, similar to those of osteosarcoma, yet avoid malignant transformation through high apoptosis rates and positive selection for p53. Notably, the antlers have evolved a highly apoptotic mechanism within the reserve mesenchyme. Moreover, deer exhibit strong positive selection for potent tumor suppressor genes such as p53. Additionally, reserve mesenchyme cells and their exosomes have demonstrated positive anti-tumor effects in both *in vitro* and *in vivo* experiments in multiple cancer models; however, the precise molecular mechanisms require further investigation.^200^

Consequently, further investigation is required regarding targets affecting mitochondria and oxidative phosphorylation. LETM1 participates in maintaining mitochondrial morphology and is closely associated with cancer stemness. Shi et al demonstrated that its overexpression in MG63/143B cells led to mitochondrial dysfunction, while its knockout inhibited the PPP and promoted oxidative phosphorylation and osteogenic differentiation.^141^ POLRMT, a mitochondrial RNA polymerase, plays a crucial role in the transcription of mitochondrial genes encoding components of the OXPHOS process. Han et al discovered elevated POLRMT transcript levels in osteosarcoma tissues. However, its shRNA suppressed U2OS xenograft growth and significantly reduced mitochondrial DNA, transcript, and respiratory chain complex expression, suggesting its critical regulatory role in OXPHOS in osteosarcoma.^201^

### Targeting other metabolic pathways

Research on the metabolic regulation of osteosarcoma has achieved a series of breakthroughs in targeting glutamine-dependent and nucleotide synthesis pathways. For instance, Ren et al used 13C-glutamine tracing to confirm that the proliferation of highly metastatic osteosarcoma cells depends on glutamine metabolism. The combination of GLS-1 inhibitors and metformin significantly suppressed tumor growth and metastasis. Additionally, nuclear magnetic resonance spectroscopy analysis revealed that this combination therapy blocked the metabolic pathway converting glutamine into TCA cycle intermediates, thereby offering a novel direction for clinical translation.^202^ Complementing this mechanism, D'Alessandro et al discovered that tumor protein p73 alpha (TAp73α) promoted mitochondrial activity, enhanced glutathione homeostasis, regulated arginine-citrulline-NO metabolism, and activated the PPP in SAOS-2 cells to accumulate NADPH by accumulating metabolic intermediates and up-regulating TCA cycle-related proteins. This revealed the multifaceted role of p53 family members in metabolic reprogramming.^203^ Additionally, Jing et al revealed that TKT directly bound to amino acid residues 201-228 of the YY1 protein, enhancing the binding capacity of YY1 to the PAK4 promoter and thereby activating the PI3K–Akt signaling pathway. Based on this mechanism, the team designed a YY1-derived peptide (YY1-PEP) segment that demonstrated anti-osteosarcoma activity *in vitro* and *in vivo*, providing a molecular tool for targeting this pathway.^204^ Wang et al further expanded our understanding of PPP regulatory mechanisms. They discovered that OR3A4 was highly expressed in osteosarcoma and correlated with disease progression. OR3A4 knockout resulted in miR-1207-5p inhibition and reduced G6PD expression, thereby decreasing NADPH production, glucose consumption, and lactate generation. This highlights the critical role of the OR3A4–G6PD axis in the PPP.^168^ Furthermore, bioinformatics analysis of the GEO dataset GSE32395 revealed significant enrichment of mitosis-related genes, such as CENPE, PRC1, TTK, and PLK4, in metabolic pathways involving PPP, providing important omics clues for identifying potential therapeutic targets.^205^

Among the intervention strategies targeting glutamine metabolism, a study examining the limitations of eflornithine (also known as difluoromethylornithine) monotherapy revealed that eflornithine treatment counteracted its anti-cancer effects by promoting nuclear translocation through the inhibition of YAP1 phosphorylation, thereby activating YAP1-mediated compensatory glutamine metabolism pathways and reducing ROS levels. Conversely, co-administration with the glutaminase inhibitor telaglenastat (CB-839) or the YAP1 inhibitor CIL56 effectively blocked this escape mechanism, providing a favorable experimental basis for developing combination therapy strategies to overcome eflornithine resistance.^206^ Meanwhile, Zhou et al confirmed that miRNA-141-3p was significantly down-regulated as a tumor suppressor in osteosarcoma. Overexpression of miR-141-3p inhibited osteosarcoma cell growth and enhanced tumor cell sensitivity to platinum-based drugs, such as cisplatin, by directly targeting GLS, further highlighting the potential value of non-coding RNAs in targeted metabolic reprogramming therapies.^167^ In summary, by directly inhibiting key metabolic enzymes to regulate upstream transcription factors and multi-level non-coding RNA networks, these studies progressively reveal the complex metabolic defense mechanisms involved in osteosarcoma. Consequently, precisely blocking these compensatory escape pathways, particularly through multilevel interventions to eliminate functional redundancy at critical metabolic nodes, is pivotal for overcoming therapeutic resistance in osteosarcoma. Current intervention strategies targeting glucose metabolism primarily encompass three categories: inhibition of glycolysis, modulation of mitochondrial function, and targeting other metabolic pathways. The core molecular targets, mechanisms of action, and anti-tumor effects are shown in Table 2.

**Table 2:** Preclinical intervention strategies targeting glycometabolism in osteosarcoma and their molecular mechanisms.

Intervention strategy	Target/mechanism	Primary effect	Model	Reference
Inhibition of glycolysis				
Inhibit P2RX7	Reduces c-Myc stability and inhibits glycolysis	Inhibits cell progression and glycolysis, reduces proliferation and metastasis	MNNG/HOS/U-2OS cells	^111^
Caudatin	Inhibits glycolysis through the Wnt/β-catenin pathway and reduces the expression of β-catenin, CyclinD1, C-myc, HK2, and LDHA	Inhibits proliferation, invasion, and glycolysis, promotes apoptosis	U2OS/MG63 cells; nude mouse model	^123^
Overexpression of miR-324-3p	Negatively regulates PGAM1 expression and inhibits PGAM1-mediated aerobic glycolysis	Inhibits cell progression and the Warburg effect; reduces proliferation, migration, and invasion	U-2OS/143B cells	^159^
Inhibit circ_0004674	Regulation of glycolysis through the miR-140-3p/TCF4 pathway, circ_0004674 acts as a miRNA sponge to up-regulate TCF4	Inhibits cell proliferation, migration, and invasion; promotes apoptosis; reduces glucose uptake and lactate production	MG63/U-2OS cells	^179^
Hydroxysafflor yellow A	Inducing ferroptosis through the HIF-1α/HK2 and SLC7A11 pathways to suppress the Warburg effect	Inhibits cell proliferation and metastasis, induces iron death, and enhances caspase-3/9 activity	MG63 cells	^187^
Pramlintide	Down-regulation of HIF-1α, up-regulation of IAPP expression, and inhibition of glycolysis	Inhibits glycolysis, reduces cell growth, and increases apoptosis	p53-deficient osteosarcoma model	^188^
GLU-MTX	Inhibition of DHFR by intracellular release of MTX through selective uptake by the GLUT1 transporter	Selectively inhibits cancer cell growth and reduces toxicity to normal cells	MG63/U-2OS/143B cells	^189^
Solasonine (SS)	Regulates glucose metabolism and inhibits glycolysis via the Wnt/β-catenin/Snail pathway	Inhibits cancer stemness and metastasis, reduces glucose uptake and lactate production	HOS/U2OS cells; nude mouse model	^190^
Overexpression of SLC9A2	ETS1 transcription inhibits SLC9A2 expression; overexpression of SLC9A2 inhibits aerobic glycolysis	Inhibits cell proliferation, invasion, and glycolysis; decreases ECAR and increases OCR	MG63 cells/nude mouse xenograft model	^191^
CDDO-NFM(20uM)	Degradation of c-MYC and inhibition of key glycolytic enzymes (PKM2, GLUT1, HK2, etc.) expression	Inhibits cell growth, reduces glucose uptake, lactate production, and ATP production	U-2OS/143B cells; nude mouse model	^192^
Vitamin C (800 μM) combine ATO (1 μM)	Synergistically inhibits PFK/PGK1/LDHA; pro-apoptotic	Enhances ATO sensitivity, inhibits proliferation, migration, and invasion, promotes apoptosis, and reduces pyruvate/lactate/ATP production	143B/U2OS cells; nude mouse model	^193^
Mitochondrial pathway				
Inhibit LETM1	Inhibition of the pentose phosphate pathway to promote oxidative phosphorylation and osteogenic differentiation	Inhibits malignant biological behaviors (proliferation, migration, invasion)	MG63/143B cells	^141^
Fructose-coated AgÅPs (F-AgÅPs)	Inhibits PDK, converting glycolysis to mitochondrial oxidative phosphorylation	Inhibits tumor growth, improves survival, and reduces osteolysis and lung metastasis	Osteosarcoma nude mouse model	^195^
Demethylzeylasteral (DEM)	Targets METTL17 and impairs OXPHOS, leading to ATP depletion	Inhibits OS growth; METTL17 overexpression reverses DEM action and restores ATP production	Osteosarcoma cells and nude mouse model	^196^
Pharmacological vitamin C	Triggers calcium release via inositol 1,4,5-trisphosphate receptors (IP3Rs), leading to mitochondrial ROS production	Induces cancer cell death	Osteosarcoma cell model	^197^
Manoalide	Leads to ROS overproduction, destroys antioxidant proteins, and activates caspase-9/-3 and PARP	Inhibits MG63 cell activity to 45.13%–4.40% and induces mitochondrial dysfunction and apoptosis	MG63 cells	^198^
Vitamin D (3) (1,25(OH)2D)	Targets VDR and reduces mitochondrial ROS/membrane potential; DDIT4/REDD1 cytoplasmic translocation inhibits mTORC1 and promotes non-oxidative metabolism	Restores redox homeostasis and inhibits tumor proliferation	MG63 osteosarcoma cells	^199^
Deer antler RM Cells/Exosomes	High apoptosis rate with positive p53 selection to avoid carcinogenesis	Shows positive anti-tumor effects in cancer models	Multiple cancer models (*in vitro*/*in vivo*)	^200^
Inhibit POLRMT shRNA	Reduced mitochondrial DNA, transcript, and respiratory chain complex expression	Inhibits U2OS xenograft tumor growth	U2OS xenograft tumor	^201^
Other avenues				
Overexpression of miR-141-3p	Targeting GLS to inhibit glutamine metabolism	Enhances cisplatin sensitivity, inhibit cell proliferation	Saos-2/U2OS cell line	^167^
Knockout of OR3A4	Up-regulates miR-1207-5p to inhibit G6PD expression	Decreases NADPH production, reduce glycolysis	MG-63/SaoS-2 cell line	^168^
GLS1 inhibitor CB-839 combined with metformin	Blocks glutamine catabolism as a tricarboxylic acid cycle intermediate	Inhibits primary focus growth and lung metastasis	Patient-derived xenograft/cell line-derived xenograft models	^202^
Activate TAp73α	Promotes mitochondrial activity and enhances NADPH production in the PPP pathway	Accumulation of metabolic intermediates and regulation of redox homeostasis	SAOS-2 cell line	^203^
YY1-PEP	Blocking TKT binding to YY1(201-228aa)	Inhibits the activation of the PI3K-Akt signaling pathway	Subcutaneous transplantation tumor model in nude mice	^204^
Eflornithine combined with YAP1 inhibitor CIL56	Blocking the YAP1-mediated glutamine metabolism compensation pathway	Increases ROS level, inhibit tumor growth	Patient-derived xenograft model	^206^

## Discussion and prospects

The malignant progression of osteosarcoma is inextricably linked to its unique reprogramming of glucose metabolism, which is centered on a multidimensional regulatory network. This consists of aberrantly expressed key enzymes, such as HK2, PKM2, LDHA, regulation by epigenetic modification-driven KDM6B, non-coding RNA network-mediated stabilization of HK2/GPI/PGK1 by circ-CTNNB1, mitochondrial dysfunction leading to SDH/COX abnormality and succinate and ROS accumulation, and c-Myc-driven synergistic PPP and glutamine metabolism, constituting a multidimensional regulatory network. This network provides energy and synthetic precursors for malignant proliferation of tumor cells, inhibits apoptosis by blocking cytochrome c release via HK2 binding to VDAC, enhances invasion and metastasis via the KDM6B-LDHA axis, induces M2 macrophage polarization via activation of the ST3GAL4/Siglec-7/9 axis by lactate accumulation, and inhibits the immune response via the delivery of miR-1246 by exosomes to remodel the immunosuppressive microenvironment. Additionally, it mediates chemoresistance via mechanisms such as simultaneous activation of autophagy and metabolic plasticity by the CLTC–VMP1 fusion gene and enhancement of DNA repair by lactate modification. Preclinical studies have shown that precise strategies targeting the key nodes of glucose metabolism have significant potential. For example, the small-molecule inhibitor CDDO-NFM inhibits HK2 and PKM2 by degrading c-Myc, DEM targets the mitochondrial translational regulator METTL17 to disrupt oxidative phosphorylation-induced ATP depletion, and gene regulatory strategies, such as silencing circ-CTNNB1 to disrupt its mediated m^6^A-HK2/GPI/PGK1 axis and overexpression of PGK1 to enhance DNA repair, overexpression of miR-324-3p to inhibit PGAM1, and smart delivery systems such as fructose-modified silver nanoparticles to redirect glycolysis to mitochondrial apoptosis by inhibiting PDK.

However, clinical translation faces several challenges. Metabolic heterogeneity is manifested by differences in the dominant pathways in different pathological settings, such as c-Myc overexpression models dependent on the P2RX7/c-Myc-HK2 axis, metastatic foci with a preference for glutamine metabolism, and drug-resistant cells that activate CLTC–VMP1-mediated dual activation of glycolysis and oxidative phosphorylation, which require single-cell multi-logical patient stratification to identify specific metabolic weaknesses. The challenge of target selectivity lies in the physiological function of targets, such as HK2 and PKM2, which may cause dose-limiting toxicities, such as myelosuppression, and require the development of tumor microenvironment-responsive smart delivery systems to enhance specificity. Compensatory resistance is particularly prominent, where inhibition of a single node, such as blocking HK2, activates YAP1-mediated glutamine compensation, and inhibition of HIF-1α is compensated for by its ligand circ_0000566 by stabilizing the HIF-1α protein. Overcoming strategies need to focus on multi-nodal synergistic interventions: combining metabolic and epigenetic regulation such as LDHA inhibitors combined with targeting the m^6^A modifier RBM15 to disrupt the HIF-1α/circ positive feedback loop, or integrating metabolic and immunological therapies such as glycolysis inhibitors combined with PD-1/PD-L1 inhibitors to reverse the lactate/GPR132 axis-mediated T-cell depletion and PD-L1 and HAVCR2 expression, or direct blockade of key escape pathways such as eflornithine in combination with the YAP1 inhibitor CIL56 or the glutaminase inhibitor CB-839.

A breakthrough lies in the integration of innovative technologies and clinical translations. Using patient-derived organoids to mimic *in vivo* metabolic dynamics and immune microenvironments, we can achieve efficacy assessments based on biomarkers such as lactate levels or specific non-coding RNAs, accelerating the translation of laboratory discoveries to the clinic. Artificial intelligence-driven multimodal data fusion can optimize individualized therapeutic decisions. Meanwhile, new targets of metabolic–immune interactions, such as the lactate/GPR132 axis and the role of succinate/SUCNR1 signaling in T-cell depletion, need to be explored in depth, and novel immunotherapies targeting metabolites or their receptors, such as combined CAR-T cells, need to be developed for metabolic plasticity, such as CLTC–VMP1-mediated switching of the energy pathway, dual-pathway inhibitors, or inducers of energy stress need to be designed. In conclusion, the study of osteosarcoma glucose metabolism is moving from mechanism analysis to precision intervention and is expected to overcome the current therapeutic bottlenecks through the integration of metabolic typing, intelligent delivery, biomarker guidance, and multimodal synergistic therapies, of which the synergy between metabolic intervention and immunotherapy will become the most promising research focus.

## CRediT authorship contribution statement

**Xuewen Ni:** Writing – original draft, Visualization, Investigation. **Ping Xia:** Writing – original draft, Methodology, Conceptualization. **Mengxue Liang:** Writing – review & editing. **Li Zhang:** Supervision, Resources. **Jiaqi Chen:** Investigation, Data curation. **Zhuojia He:** Writing – review & editing, Validation. **Hua Huang:** Writing – review & editing, Formal analysis. **Jing Feng:** Writing – review & editing, Project administration. **Xun Niu:** Data curation. **Feifei Pu:** Writing – review & editing, Supervision, Funding acquisition, Conceptualization.

## Funding

This work was supported by the 10.13039/501100001809National Natural Science Foundation of China (No. 82474545, 82274559), 10.13039/501100003819Hubei Provincial Natural Science Foundation of China (No. 2024AFB1011), 10.13039/501100002858China Postdoctoral Science Foundation (No. 2024T170247, 2024M750820), the Research Project of Traditional Chinese Medicine of Hubei Provincial Administration of Traditional Chinese Medicine (China) (No. ZY2025Q039), the Natural Science Foundation of Wuhan (China) (No. 2024040801020366).

## Conflict of interests

The authors declare that they have no conflict of interests.
